# Effects of Individualized Treadmill Endurance Training on Oxidative Stress in Skeletal Muscles of Transgenic Sickle Mice

**DOI:** 10.1155/2019/3765643

**Published:** 2019-07-24

**Authors:** Etienne Gouraud, Emmanuelle Charrin, John J. Dubé, Solomon F. Ofori-Acquah, Cyril Martin, Sarah Skinner, Benjamin Chatel, Anaelle Boreau, Laurent A. Messonnier, Philippe Connes, Vincent Pialoux, Christophe Hautier, Camille Faes

**Affiliations:** ^1^Interuniversity Laboratory of Human Movement Biology EA7424, Vascular Biology and Red Blood Cell Team, University Claude Bernard Lyon 1, Villeurbanne, France; ^2^Laboratory of Excellence “GR-Ex”, Paris, France; ^3^Division of Hematology/Oncology, Department of Medicine, University of Pittsburgh, Pittsburgh, PA 15261, USA; ^4^Division of Endocrinology and Metabolism, University of Pittsburgh, Pittsburgh, PA 15261, USA; ^5^Department of Biology, Chatham University, Pittsburgh, PA 15232, USA; ^6^Center for Translational and International Hematology, Vascular Medicine Institute, University of Pittsburgh, Pittsburgh, PA 15261, USA; ^7^Interuniversity Laboratory of Human Movement Biology EA7424, Vascular Biology and Red Blood Cell Team, University Savoie Mont Blanc, Chambéry, France; ^8^Institut Universitaire de France, Paris, France

## Abstract

Oxidative stress is a key feature in the pathophysiology of sickle cell disease. Endurance training has been shown to reduce oxidative stress in the heart and the liver of sickle mice. However, the effects of endurance training on skeletal muscles, which are major producers of reactive oxygen species during exercise, are currently unknown. The aim of this study was to evaluate the effect of sickle genotype on prooxidant/antioxidant response to individualized endurance training in skeletal muscles of sickle mice. Healthy and homozygous Townes sickle mice were divided into trained or sedentary groups. Maximal aerobic speed and V̇O_2_ peak were determined using an incremental test on a treadmill. Trained mice ran at 40% to 60% of maximal aerobic speed, 1 h/day, 5 days/week for 8 weeks. Oxidative stress markers, prooxidant/antioxidant response, and citrate synthase enzyme activities were assessed in the *gastrocnemius*, in the *plantaris*, and in the *soleus* muscles. Maximal aerobic speed and V̇O_2_ peak were significantly reduced in sickle compared to healthy mice (-57% and -17%; *p* < 0.001). NADPH oxidase, superoxide dismutase, and catalase activities significantly increased after training in the *gastrocnemius* of sickle mice only. A similar trend was observed for citrate synthase activity in sickle mice (*p* = 0.06). In this study, we showed an adaptive response to individualized endurance training on the prooxidant/antioxidant balance in the *gastrocnemius,* but neither in the *plantaris* nor in the *soleus* of trained sickle mice, suggesting an effect of sickle genotype on skeletal muscle response to endurance treadmill training.

## 1. Introduction

Sickle cell disease (SCD) is a genetic disorder characterized by hemolytic anemia and vasoocclusive crises [1] resulting in an enhanced production of reactive oxygen species (ROS) [2, 3]. In this context, higher levels of markers of oxidative stress have been found in the plasma and RBC of patients with SCD [4], as well as in the liver, kidney, and spleen of transgenic sickle mice [5].

Little research has been conducted to understand the pathophysiology of skeletal muscles in SCD, but recently, several studies started to report functional and histological alterations in skeletal muscle [6–9]. In SCD patients at rest, Ravelojaona et al. [8] observed skeletal muscle microvasculature remodeling, amyotrophy, and decreased activity of key enzymes involved in energy metabolism, including creatine kinase (CK), which could be explained by an excessive ROS content in the skeletal muscle of SCD patients [10]. Additionally, recent studies suggest that increased oxidative stress may impair force production in transgenic sickle mice during acute exercise [6] which could be attributed to the increased intramyocellular acidosis [11, 12] observed in these mice [7]. However, while it is well recognized that skeletal muscle is the major endogenous source of ROS during exercise [13, 14], little research has been done to investigate oxidative stress production in the skeletal muscle of homozygous SCD patients or mice.

While vigorous physical activity is usually not recommended for SCD patients [15], recently, a randomized controlled trial reported that an individualized and standardized moderate-intensity training program in sickle patients was clinically safe and improved the functional capacity and the skeletal muscle characteristics of those patients [16]. Besides, chronic exercise in two transgenic sickle mice models [17, 18] was reported to decrease oxidative stress and inflammation in several organs. In SAD mice, voluntary wheel running protocol decreased lipid peroxidation in the heart after hypoxia/reoxygenation stress [17]. In Townes mice, an endurance treadmill running protocol decreased oxidative stress in the heart and the liver and attenuated systemic inflammation [18]. However, Chatel et al. [19] reported that such training did not induce muscular oxidative stress in Townes mice. Nevertheless, the running speed was not individualized for each mouse that may explain the lack of significant changes in Chatel et al.'s study. From a clinical point of view, the individualization of the exercise in sickle cell disease patients is of primary importance in the training management of these patients.

Thus, we chose to investigate the effects of 8-week individualized moderate-intensity treadmill training program on the oxidative stress levels in the skeletal muscle of healthy and transgenic Townes sickle mice. The main hypothesis of our work is that sickle genotype affects the prooxidant/antioxidant response in the skeletal muscle of Townes mice subjected to such endurance training program. We chose the *gastrocnemius*, the *plantaris*, and the *soleus* in this study for their specific typology and involvement in treadmill locomotion.

## 2. Material and Methods

### 2.1. Mice

The study was approved by the Institutional Animal Care and Use Committee (IACUC) at the University of Pittsburgh (protocol #13102567). Eight-week-old male Townes mice were used in this study. Townes mice were obtained by establishing a colony using breeding pairs purchased from Jackson Laboratory (Bar Harbor, ME, USA). Mice were provided with food and water ad libitum and maintained on a 12-hour light-dark cycle.

### 2.2. Incremental Treadmill Test

Healthy AA (*n* = 12) and sickle SS (*n* = 17) mice were progressively acclimatized to treadmill exposure by increasing intensity and duration for 3 days. For the incremental test, mice ran on an enclosed, single lane treadmill (molecular enclosed metabolic treadmill for mice; Columbus Instruments), and real-time measurements of oxygen consumption (V̇O_2_) and carbon dioxide output (V̇CO_2_) were performed using an Oxymax/Comprehensive Laboratory Animal Monitoring System (CLAMS; Columbus Instruments). Mice ran at 5, 9, 12, and 15 m·min^−1^ at a 15° inclination for 5 min at each velocity [20]. Treadmill velocity was then increased by 2 m·min^−1^ every 2 min until exhaustion, defined as the inability to return to treadmill running after 10 seconds. Maximal aerobic speed (MAS) was defined as the speed at which V̇O_2_ plateaued. We chose to do not assess V̇O_2_ posttraining to keep enough mice in each group of mice and to avoid bias analysis. Indeed, the risk of death of the Townes mice during this kind of incremental maximal exercise test dramatically increases with age according to the progression of disease severity [21].

### 2.3. Individualized Treadmill Aerobic Training Protocol

**Figure 1:**
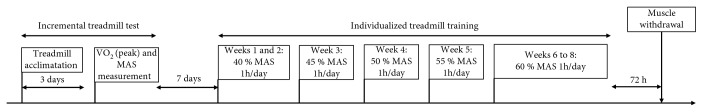
Schematic view of the training protocol procedure for healthy and sickle trained mice. V̇O_2_ (peak): peak oxygen consumption; MAS: maximal aerobic speed.

After 7 days of rest, they were randomly distributed into 4 groups: trained AA (Tr-AA), sedentary AA (Sed-AA), trained SS (Tr-SS), and sedentary SS (Sed-SS). Trained mice ran 1 h/day, 5 days/week at 40%-60% MAS for 8 weeks at 15° inclination on a motorized treadmill (Figure 1) [22] (RM Exer-3/6 open treadmill with manual incline; Columbus Instruments). If mice did not keep up with treadmill speed, they were manually encouraged to run and/or exposed to brief periods of electric shock. The sedentary groups were exposed to the treadmill 1 day/week and handled daily in the same way as mice in the trained group.

### 2.4. Muscle Sampling

Mice were anesthetized and euthanized by cervical dislocation 72 h after the last exercise session. The *gastrocnemius*, *soleus*, and *plantaris* were harvested, weighed, and then immediately frozen in liquid nitrogen. Muscles were homogenized in a lysis buffer (20 mM Tris; 1 mM ethylenediaminetetraacetic acid; 100 mM sodium chloride; 0.5% (*v*/*v*) Triton x100) and centrifuged at 12 000 RCF and 4°C for 10 min. Protein concentrations were assayed with a BCA kit (Novagen #71285.3, Darmstadt, Germany), and all results were normalized by grams of total protein.

### 2.5. Oxidative Stress and Antioxidant Enzyme Activities in Skeletal Muscle

All biochemical products used for oxidative stress assays were purchased from Sigma-Aldrich (St. Louis, MO, USA), and spectrophotometric measurements were performed on a TECAN Infinite 2000 plate reader (Männedorf, Switzerland).

#### 2.5.1. Oxidative Stress Markers

Muscle malondialdehyde (MDA) concentrations were determined as thiobarbituric reactive substances, as previously described [23]. NADPH oxidase (NOX) and xanthine oxidase (XO) activities were calculated by measuring the kinetic of appearance of the complex superoxide anion/nitrotetrazolium blue (NTB) spectrophotometrically at 560 nm [24].

#### 2.5.2. Antioxidant Enzymes

Glutathione peroxidase (GPX) activity in skeletal muscle was assayed using Paglia and Valentine's [25] modified method, which uses hydrogen peroxide (H_2_O_2_) as a substrate. Superoxide dismutase (SOD) activity was quantified using the Beauchamp and Fridovich's [26] method, slightly modified by Oberley and Spitz [27]. Catalase (Cat) activity in the skeletal muscle was determined using Johansson and Borg's [28] method, which uses H_2_O_2_ as a substrate and formaldehyde as a standard.

#### 2.5.3. Citrate Synthase Activity

Citrate synthase (CS) activity was assessed following the production of mercaptide ions spectrophotometrically at 412 nm by adding dinitrothiocyanobenzene (DTNB), acetyl-CoA, and oxaloacetic acid.

### 2.6. Statistical Analysis

Data are expressed as the mean ± SEM. Statistical analyses were performed using GraphPad Prism 6 (GraphPad Software, La Jolla, CA, USA). Normality was checked using the Kolmogorov-Smirnov test. Student's *t*-test was used to compare exercise capacity between healthy and sickle mice. Two-way ANOVA followed by Tukey's post hoc test was used to compare enzyme activities and oxidative stress markers among groups. The significance level was set at *p* < 0.05. A tendency was also considered for 0.05 ≤ *p* < 0.1. The statistical power (*β*) has been calculated with and alpha level set at 0.05.

## 3. Results

### 3.1. Peak Exercise Capacity

At baseline, incremental treadmill tests showed that SS mice had 17% lower V̇O_2_ peak than AA mice (Table 1). Also, MAS was significantly decreased in SS mice compared to AA mice (-57%; Table 1).

### 3.2. Citrate Synthase Activity

**Figure 2:**
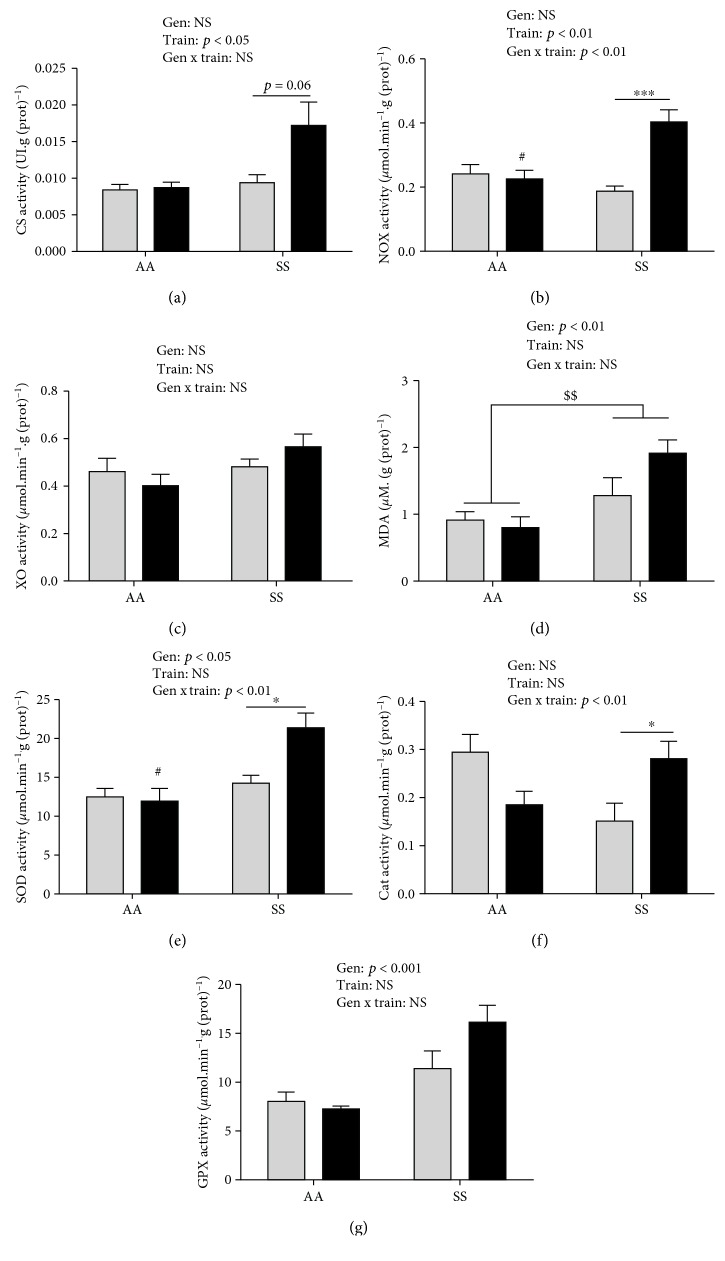
Citrate synthase (CS) activity (a), prooxidant enzyme activities (b, c) malondialdehyde (MDA) concentration (d), and antioxidant enzyme activities (e–g) in the *gastrocnemius*. Cat: catalase; GPX: glutathione peroxidase; NOX: NADPH oxidase; SOD: superoxide dismutase; XO: xanthine oxidase. Data are expressed as the mean ± SEM. Gen: genotype effect; Train: training effect; Gen x train: genotype × interaction effect. ^∗^*p* < 0.05, ^∗∗∗^*p* < 0.001, ^$$^*p* < 0.01, and ^#^*p* < 0.01*vs.* Tr-SS. *n*(Sed‐AA) = 6, *n*(Tr‐AA) = 6, *n*(Sed‐SS) = 7, and *n*(Tr‐SS) = 10. Grey bars: sedentary mice; black bars: trained mice.

In the *gastrocnemius*, CS activity was significantly higher in SS compared to AA mice in the trained group (*p* < 0.05, *β* = 0.92). It tended to be higher in trained SS compared to their sedentary counterparts (*p* = 0.06, *β* = 0.81, Figure 2(a)) while no differences were observed between Tr-AA and Sed-AA mice (*p* = 0.99, *β* = 0.06, Figure 2(a)).

In the *plantaris* and the *soleus*, CS activity did not differ between the four groups.

### 3.3. Prooxidant and Oxidative Stress Markers

In the *gastrocnemius*, a significant interaction effect (genotype × training, *p* < 0.01, *β* = 0.99) was observed for NOX while neither genotype nor training effects were identified for XO activity (Figures 2(b) and 2(c)). NOX activity was 2-fold higher (*p* < 0.001, *β* = 1) in trained SS compared to their sedentary counterparts (Figure 2(b)). Training did not significantly change MDA in healthy or sickle mice. However, a significant genotype effect (*p* < 0.01) was observed for MDA with a higher level of MDA observed in SS compared to AA mice, as evidenced by an almost twofold higher level in Tr-SS compared with Tr-AA mice (Figure 2(d)).

**Figure 3:**
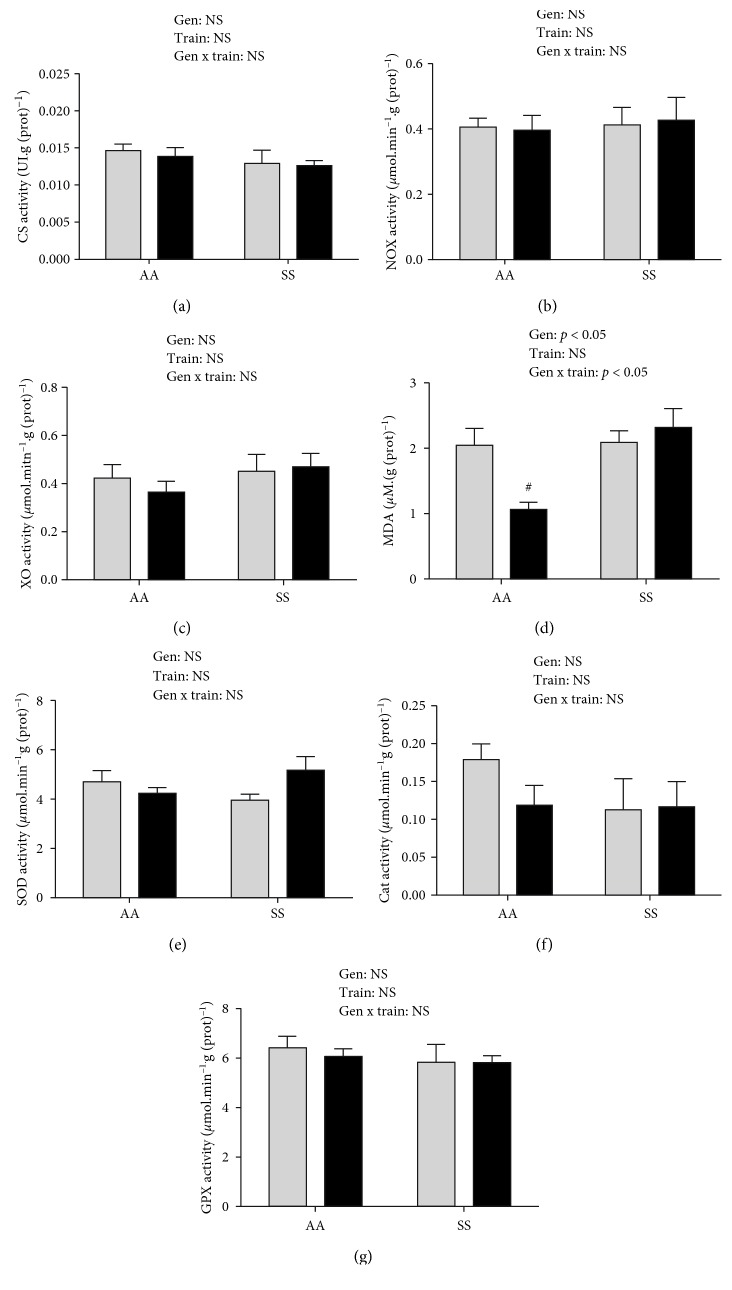
Citrate synthase (CS) activity (a), prooxidant enzyme activities (b, c) malondialdehyde (MDA) concentration (d), and antioxidant enzyme activities (e–g) in the *plantaris*. Cat: catalase; GPX: glutathione peroxidase; NOX: NADPH oxidase; SOD: superoxide dismutase; XO: xanthine oxidase. Data are expressed as the mean ± SEM. Gen: genotype effect; Train: training effect; Gen x train: genotype × interaction effect. ^#^*p* < 0.05*vs.* Tr-SS. *n*(Sed‐AA) = 6, *n*(Tr‐AA) = 6, *n*(Sed‐SS) = 7, and *n*(Tr‐SS) = 10. Grey bars: sedentary mice; black bars: trained mice.

In the *plantaris*, XO and NOX activities were not modified by training in the AA or SS mice (Figures 3(b) and 3(c)). In contrast, MDA concentrations were significantly higher in Tr-SS *vs.* Tr-AA mice (*p* < 0.05, *β* = 1, Figure 3(d)) and were twofold lower in trained *vs.* sedentary AA mice.

**Figure 4:**
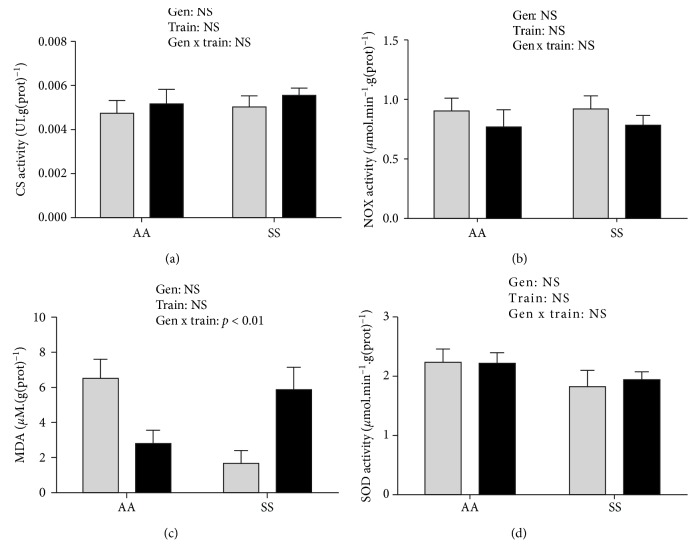
Citrate synthase (CS) activity (a), NADPH oxidase (NOX) activity (b), malondialdehyde (MDA) concentration (c), and superoxide dismutase (SOD) activity (d) in the *soleus*. Data are expressed as the mean ± SEM. Gen: genotype effect; Train: training effect; Gen x train: genotype × interaction effect. *n*(Sed‐AA) = 6, *n*(Tr‐AA) = 6, *n*(Sed‐SS) = 7, and *n*(Tr‐SS) = 10. Grey bars: sedentary mice; black bars: trained mice.

In the *soleus*, MDA concentrations did not differ between the four groups. However, similar to the *plantaris*, a significant interaction effect (genotype × training, *p* < 0.01, *β* = 0.72) was observed with a nearly twofold higher level in Tr-SS compared to Tr-AA mice (Figure 4(c)).

### 3.4. Antioxidant Enzyme Activities

In the *gastrocnemius* of SS mice, we found significantly higher SOD (*p* < 0.05, *β* = 0.96, Figure 2(e)) and catalase activities (*p* < 0.05, *β* = 0.82, Figure 2(f)) (50% and 85%, respectively) in Tr-SS compared to Sed-SS mice. In addition, significantly higher SOD activity was found in Tr-SS compared to Tr-AA mice (*p* < 0.05, *β* = 0.99, Figure 2(e)). Training did not significantly affect GPX activity in Tr-SS compared to Sed-SS mice (*p* = 0.12, *β* = 0.57, Figure 2(g)). However, a genotype effect was identified (*p* < 0.01) with higher GPX in SS compared to AA mice independent of a sedentary or trained status.

In the *plantaris* and in the *soleus*, neither training nor genotype effects were observed (Figures 3(e)–3(g); Figure 4(d)).

## 4. Discussion

The aim of this study was to evaluate the effects of sickle genotype on the prooxidant/antioxidant response to endurance exercise in three skeletal muscles of SCD transgenic mice. Our results demonstrated that an 8-week individualized moderate-intensity treadmill training program (i) increased oxidative stress markers and (ii) increased antioxidant enzyme activity in the *gastrocnemius*, but not in the *plantaris* and in the *soleus* of trained Townes SS mice.

Markers of lipid peroxidation and prooxidant enzymes activities are higher in SS mice after 8 weeks of individualized moderate-intensity treadmill training program. Indeed, NOX [29] was reported as a main producer of ROS in skeletal muscle, and CS [30] has been extensively used as a marker for assessing muscle mitochondrial volume density as well as mitochondrial ROS production. Both may indicate higher ROS production in the *gastrocnemius*. Also, elevated intracellular acidosis previously observed in these mice [7] may cause higher ROS production. Acidosis has been shown to increase oxidative stress by promoting Fenton reactions and by causing a protonation of the peroxynitrite anion that can lead to the production of radical hydroxyl, one of the most powerful ROS [11, 12]. Meanwhile, the 8-week individualized moderate-intensity treadmill training program also increased antioxidant enzymes. This suggests that the pro-antioxidant balance in the *gastrocnemius* of trained sickle mice could be equilibrated as illustrated by the lack of change in MDA in response to the exercise training. Our findings are in accordance with previous studies in rodents after aerobic training [31–33], as we found higher SOD and catalase activities in trained sickle mice. This increase of antioxidant enzyme activities could be attributed to the increase in NOX activity and subsequent ROS production involved in the oxidative stress signaling pathway [33, 34]. Higher CS activity may also be explained by an increased ROS production which could act as a signaling stimulus to increase mitochondrial content and oxidative capacity in skeletal muscles [35]. As previously reported in healthy mice, exogenous supplementation of N-acetylcysteine, a precursor of glutathione synthesis, was found to attenuate exercise-induced upregulation of endogenous antioxidants, including SOD and catalase activities in the *gastrocnemius* [33]. Those results suggest [33] the importance of a minimal ROS content (i.e., mild prooxidant environment) to training-induced adaptation of skeletal muscle.

While endurance training has been found to reduce inflammation, splenic enlargement, cardiac and hepatic oxidative stress in a similar transgenic sickle mice model [18], these results suggest an adaptive response to endurance training on pro-antioxidant balance in the skeletal muscle of sickle mice. Surprisingly, resting NOX and XO activities as well as MDA concentration did not differ between AA and SS sedentary mice. Therefore, it is likely that oxidative stress is not changed at rest in skeletal muscle in sedentary sickle mice, unlike in their trained counterparts. This could also suggest that skeletal muscle may be less affected by the disease than other organs in these mice [5].

Changes in the balance of pro-antioxidant markers in the *gastrocnemius* after an 8-week individualized moderate-intensity treadmill training program were only observed in trained SS mice and not in their AA counterparts. Previous results reported no modifications in muscular oxidative stress in AA mice exposed to endurance training program [19] while higher training intensity was shown to decrease oxidative stress and increase antioxidant markers in the muscle of healthy mice [36]. Therefore, our training intensity/speed (40%-60% MAS) could not be enough to trigger oxidative stress adaptations in the *gastrocnemius* of AA Townes mice. In addition, our results in the *gastrocnemius* differ from those of Chatel et al. [19], who did not report changes in the pro-antioxidant balance between trained and sedentary sickle mice in the *tibialis anterior* after 7 weeks of aerobic training. These differences may be explained by the individualization of the training speed in our study (speed calculated from the percentage of MAS from each mice) compared to Chatel et al. [19] who used the same speed for all the mice or by the muscle investigated (i.e., *tibialis anterior*), whose typology is nearly similar to the *plantaris* [37].

While moderate endurance training induced oxidative stress adaptations in the *gastrocnemius* of Tr-SS, no effects were observed in the *plantaris* and the *soleus*. The differential response to endurance training in each muscle could be explained by their relative contribution in treadmill locomotion. Indeed, the *gastrocnemius*, which represents more than 80% of the posterior hindlimb muscles in the mice [6], has a higher contribution in the generation of mechanical work during locomotion than the *plantaris* and *soleus* [38].

Thus, it seems that the skeletal muscle work generated by running at 40-60% of MAS was sufficient to induce muscular changes in oxidative stress in Tr-SS but was too mild to trigger oxidative stress adaptations in Tr-AA mice. Therefore, our data provide evidence that the use of individualized training intensities might be a factor to consider when designing endurance training protocols for sickle cell mice or patients.

## 5. Conclusion

In conclusion, our results show that an 8-week individualized moderate-intensity treadmill training program increased prooxidant and antioxidant enzyme activities in the *gastrocnemius* of transgenic sickle mice suggesting an effect of SS genotype on pro-antioxidant response to endurance training in these mice. Individualization of exercise intervention should be considered in the context of therapeutic care of SCD patients, in line with the conclusions of the first randomized controlled trial training program performed in homozygous SCD patients [16]. Our results combined with the improvement in skeletal muscle characteristics reported in Gellen et al.'s study [16] strengthen the idea that skeletal muscle is a key target to consider in further therapeutic protocol. Further studies should be conducted to better characterize the percentage of training intensity that may trigger oxidative stress and skeletal muscle adaptations without associated complications in these individuals.

## Figures and Tables

**Table 1:** Peak exercise capacity activity for all groups prior to training.

	AA (*n* = 12)	SS (*n* = 17)
VO_2_ peak (ml·h^−1^·kg^−1^)	8407.0 ± 224.5	7046±118.2^∗∗∗^
Maximal aerobic speed (m·min^−1^)	29.3 ± 1.7	13.6±0.5^∗∗∗^

Data are expressed as mean ± SEM. ^∗∗∗^*p* < 0.001.

## Data Availability

The data used to support the findings of this study are available from the corresponding author upon request.

## Abbreviations

AA: Healthy genotype of hemoglobin
ANOVA: Analysis of variance
AS: Heterozygous genotype of sickle cell disease
Cat: Catalase
CK: Creatine kinase
COX: Cytochrome C oxidase
CS: Citrate synthase
DTNB: Dinitrothiocyanobenzene
GR: Glutathione reductase
GSH: Glutathione
GPX: Glutathione peroxidase
H_2_O_2_: Hydrogen peroxide
HbS: Hemoglobin S
MAS: Maximal aerobic speed
MDA: Malondialdehyde
NADPH: Nicotinamide adenine dinucleotide phosphate
NOX: NADPH oxidase
RBC: Red blood cell
ROS: Reactive oxygen species
SCD: Sickle cell disease
Sed-AA: Sedentary healthy Townes mice
Sed-SS: Sedentary homozygous sickle Townes mice
SOD: Superoxide dismutase
SS: Homozygous genotype of sickle cell disease
Tr-AA: Trained healthy Townes mice
Tr-SS: Trained homozygous sickle Townes mice
V̇CO_2_: Carbon dioxide output
V̇O_2_: Oxygen consumption
XO: Xanthine oxidase.
